# Testing the theory of Kuznet curve on environmental pollution during pre- and post-Covid-19 era

**DOI:** 10.1038/s41598-023-38962-5

**Published:** 2023-08-08

**Authors:** Oluwaseun Samuel Oduniyi, John M. Riveros, Sherif M. Hassan, Ferhat Çıtak

**Affiliations:** 1 Department of Agricultural and Applied Economics, Texas Tech University, Lubbock, TX USA; 2 Estudios Y Evaluación de La Gestión Pública Colombian, Colombia, USA; 3 CNMS, Marburg University, Marburg, Germany; 4 Hitit University, Çorum, Turkey

**Keywords:** Environmental social sciences, Pathogenesis

## Abstract

Covid-19 has brought about significant changes in people’s daily lives, leading to a slowdown in economic activities and the implementation of restrictions and lockdowns. As a result, there have been noticeable effects on the environment. In this study, we examine the impact of Covid-19 total cases on the monthly average of carbon monoxide emissions in developed economies known for heavy pollution, covering the period from 2014 to 2023. We apply the Ambiental Kuznets curve approach to analyze the data. By employing different panel estimation techniques such as fixed effects and Driscoll-Kraay regressions, we observe a marked shift in environmental dynamics during the post-Covid era. This shift alters the statistical significance of the N-shaped Kuznets curve, rendering the relationship between economic activity and environmental impact non-significant. Interestingly, the Covid-related variables utilized in the various estimations are not statistically significant in explaining the long-term environmental effects.

## Introduction

The environment, people's livelihoods, and the global economy have all been significantly impacted by Covid-19. Recent literature has presented conflicting findings regarding Covid-19's effects on the environment. According to data from the European Environmental Agency, the Covid-19 lockdown and expectations of a slowdown in economic activity led to a temporary decline in CO2 emissions in many European cities, including Barcelona, Madrid, Milan, Rome, and Paris^1^.

Most of the studies have postulated that the Covid-19 pandemic has improved air quality, attributing such an effect to the decreased Greenhouse Gas (GHGs) emissions. The reduced economic activity, shutting down of energy-intensive factories, suspension of air and train traffic, decline in tourism, and decrease in vehicle traffic are all primary causes of the improved air quality^2,3^. The empirical results in general confirm the reductions of pollutants in the atmosphere during Covid-19, for instance in Quito, the capital of Ecuador, there was a reduction in concentrations of SO_2_, NO_2_, and CO together with the lockdown enforcement^4^. In Eastern Europe, during the lockdown period, there was a significant reduction in CO and NO due to the decrease in transport activities^5^. Other studies enlaced the short-term improved air quality with reduced electricity production and consumption^6^. However, it is generally perceived that the improved air quality due to the pandemic contamination measures is short-termed, and is anticipated to be reversed with the gradual increase in human activity^7,8^.

Contrarily, other research has shown that during the pandemic the volumes of organic and inorganic waste have increased attributing this to (a) reduction in recycling services, (b) heavy transport, and mobilization of food production and related delivery services, (c) a sharp increase in demand for single-use plastic packaging, bottled hand sanitizer, and other personal protective equipment (PPE) worldwide, (d) increase the demand for home delivery services, and (e) increase in medical waste^9,10^.

Another stream of the literature investigates the environmental spillovers of Covid-19 considering the Environmental Kuznets Curve (EKC) theory^11^ argue that economic growth hurts the environment, while^12^ on the contrary support the claim that economic growth and environmental quality are complements^13^ demonstrate the existence of a non-linear link between economic expansion and environmental degradation, which can be depicted as a bell-shaped curve known as the Environmental Kuznets curve (EKC). Some studies found evidence of the validation of the inverted N-shaped EKC hypothesis in the long term considering the relationship between oil prices, oil rents, and Co2 in KSA^14^. Another study by^15^ confirms the likely occurrence of environmental degradation during the economic recovery post-Covid-19. During the Covid-19 pandemic, worldwide energy consumption and, as a result, environmental emissions were lowered. According to the EKC, any gain in real income can increase pollution emissions during the initial stage of economic development, therefore economic recovery from the pandemic might raise pollution emissions even higher than before the epidemic. In this study, we investigate the mitigating non-linear effects of Covid-19 on the nexus between the environment, proxied by carbon monoxide, and economic activity, proxied by the Economic Activity Index (EIA). Using a sample of the top largest economies over the period from 2014 to 2023, we separate our results into pre. and post Covid-19. Before the Covid-19 outbreak, the environment-growth nexus is best articulated by a statistically significant N-shape Kuznets curve. After the Covid-19 outbreak, these dynamics are mitigated, and the Kuznets curve is barely significant under a 90% level of confidence.

## Literature review

The majority of the research use a comparative pre-post strategy to examine the environmental impacts of Covid 19 on air gas pollutants such as carbon monoxide (CO), nitrogen oxides (NO and NO_2_), carbon dioxide (CO_2_). Table 1 summarises the important findings of selected recent research. Part A of the table contains studies at the municipal or provincial level, whereas Part B contains research at the country level.

**Table 1:** Summary of literature review.

	Authors	Sample	Key findings
*A. Studies focusing on the COVID-19 outbreak and air quality (CO*_*2*_* emissions) at the city level*			
^16^	Tian, X., An, C., Chen, Z., & Tian, Z. (2021)	Eight Canadian Cities	The impact of the Covid-19 pandemic on CO_2_ emission concentration in the atmosphere is significant in each city
^17^	Gao, C., Li, S., Liu, M., Zhang, F., Achal, V., Tu, Y., Zhang, S., & Cai, C. (2021)	Chinese megacities	Covid-19 epidemic improves air quality
^18^	Han, L., Zhao, J., & Gu, Z. (2021)	Xi'an, China	Covid-19 pandemic enhances the quality of air
^19^	Prats, R.M., van Drooge, B.L., Fernández, P., Marco, E., & Grimat, J.O. (2021)	Barcelona, Spain	A negative relationship is observed between Covid-19 pandemic and air pollution
^20^	Hashim, B.M., Al-Naseri, S.K., Al-Maliki, A., & Al-Ansari, N. (2021)	Baghdad, Iraq	Covid-19 helps in air quality and natural environment
^21^	Borhani, F., Motlagh, M.S., Stohl, A., Rashidi, Y., & Ehsani, A.H. (2021)	Tehran, Iran	Covid-19 pandemic helped reduce CO_2_ emission
^22^	Magazzino, C., Mele, M., & Schneider, N. (2020)	Three French Cities	A direct relationship between Covid-19 pandemic and air pollution have been found
^23^	Mitra, A., Chaudhuri, T.R., Mitra, A., Pramanick, P., & Zaman, S. (2020)	Kolkata City, India	Covid-19 epidemic lowers CO_2_ emission
^24^	Tello-Leal, E., & Macías-Hernández, B.A. (2020)	Victorio, Mexico	A positive correlation exists between Covid-19 pandemic and CO_2_ emission
^25^	Bashir, M.F., Bilal, B.M., Komal, B., Bashir, M.A., Tan, D., & Bashir, M. (2020)	New York City, USA	Air quality is significantly linked to changes in the Covid-19 epidemic
^26^	Zhang, Z., Xue, T., & Jin, X. (2020)	219 Chinese cities	Air pollution is positively associated with Covid-19 cases
^27^	AQOP. (2021)	355 Municipalities, Netherlands	Pollution concentration has a positive influence on confirmed Covid-19 cases
^28^	Rahman, S., Azad, A.K., Roquia Salam, H., Islam, A.R.T., Rahman, M., & Hoque, M.M. (2020)	Dhaka City, Bangladesh	Covid-19 outbreak plays an important role in air pollution
^29^	Zoran, M.A., Savatru, R.S. Savastru, D.M., & Tautan, M.N. (2020)	Milan, Italy	Covid-19 epidemic was having a positive role in air pollution reduction
^30^	Nakada, L.Y.K. & Urban, R.C. (2020)	São Paulo State, Brazil	Covid-19 pandemic has a positive impact on air quality
^31^	Hutter, H-P., Poteser, M., Moshammer, H., Lemmerer, K., Mayer, M., Weitensfelder, L., Wallner, P., & Kundi, M. (2020)	Vienna, Austria	An adverse association between air pollution and Covid-19 was deduced
^32^	Otmani, A., Benchrif, A., Tahri, M., Bounakhla, M., Chakir, E.M., El Bouch, M., & Krombi, M. (2020)	Salé City, Morocco	A significant reduction in emissions was observed during the Covid-19 pandemic
^33^	Kerimray, A., Baimatova, N., Ibragimova, O.P., Bukenov, B., Kenessov, B., Plotitsyn, P., & Karaca, F. (2020)	Almaty, Kazakhstan	Covid-19 outbreak and air pollution are strongly correlated with each other
^34^	Mahato, S., Pal, S., & Ghosh, K.G. (2020)	Delhi, India	Covid-19 pandemic is significantly improved air quality
*B. Studies focusing on Covid-19 outbreak and air quality (CO*_*2*_* emissions) at the country level*			
^2^	Filonchyk, M., Huryvonich, V., & Yan, H. (2021)	Poland	The connection between Covid-19 outbreak and air quality has been found positive
^35^	Gama, C., Relvas, H., Lopes, M., & Monteiro, A. (2021)	Portugal	Air quality improved during the pandemic
^36^	Iqbal, S., Bilal, A.R., Nurunnabi, M., Iqbal, W., Alfakhri, Y., & Iqbal. N. (2020)	Pakistan	Covid-19 pandemic lead to a negative impact on CO_2_ emissions
^37^	Mele, M., & Magazzino, C. (2020)	India	Covid-19 outbreak and CO_2_ emission is directly connected
^5^	Yusup, Y., Ramli, N.K., Kayode, J.S., Yin, C.S., Hisham, S., Isa, H.M., & Ahmad, M.I. (2020)	China, USA, Europe, and India	A negative connection exists between Covid-19 pandemic and atmospheric CO_2_ concentration
^38^	Zu, Y., Xie, J., Huang, F., & Cao, L. (2020)	China	A positive and significant relationship between Covid-19 and CO_2_ emission was found
^39^	Zambrano-Monserrate, M.A., Ruano, M.A., & Sanchez-Alcalde, L. (2020)	China, USA, Italy, and Spain	Both positive and negative indirect impacts of Covid-19 on air quality were observed
^40^	Berman, J.D. & Ebisu, K. (2020)	USA	The covid-19 pandemic declines air pollution

This study is one of the few which studied the mitigating environmental effects of Covid-19 considering the Environmental Kuznets curve. An example of these studies is the country-level study by^41^ that found evidence for the validation of the inverted N-shaped EKC hypothesis levels in the long term considering the relationship between oil prices, oil rents, and CO_2_ in KSA. By applying to a panel of countries using Global VAR, another piece of evidence by^42^ confirm the likely occurrence of environmental risk post Covid-19 economic recovery. According to the EKC, as the globe recovers, pollution emissions will grow even higher than before the outbreak. Our study is the first according to our knowledge that uses the latest data up to 2023 to test the EKC hypothesis over two temporal samples, Pre and post Covid-19.

## Methodology

Using the approach of the Ambiental Kuznets curve^43,44^ which specifies the relationship between contamination and economic growth, we will opt for a similar, yet slightly modified version where we use the monthly average carbon monoxide emissions measured in micrograms per cubic meter of air µg/m^3^ as the dependant variable following^27^. To proxy the pandemic effects, we use the monthly sums of the cases of Covid-19 by country using the information of the European Centre for Disease Prevention and Control^45^ with information augmented up to 2023 from the Johns Hopkins Center for Systems Science and Engineering^46^. To serve this purpose the model is specified as:

1 $$CO_{it} = \beta_{0} + \beta_{1} EAI_{it} + \beta_{2} EAI_{it}^{2} + \beta_{3} EAI_{it}^{3} + \alpha ^{\prime}X + \delta_{1} Cov_{it} + e_{it}$$

where $C{O}_{it}$ is the monthly average carbon monoxide emissions of country $i$ at month $t$, the monthly economic activity index by country $EA{I}_{it}$, and a set of nonlinear terms represented in $EA{I}_{it}^{2}$ and $EA{I}_{it}^{3}$ to capture marginal effects and N-type shape relationships. We include a vector of covariables represented in $X$ (see for detail the variables by country in Appendix A, supplementary Table A1) for the same monthly periodicity, in which we selected the unemployment rate, and the inflation (considering the consumer price index by country). For this research, we isolate the inclusion of the Covid-19 variable of total cases $Cov$ to inspect the statistical significance and sign of the ${\delta }_{1}$ coefficient across the estimations.

The sample period is from December 2014 to January 2023. We separate our analysis into a pre-covid before October 2019, and a post-covid analysis from October 2019 to January 2023. For this purpose, we apply unit-root tests of first and second generation (Fisher-type Augmented Dickey-Fuller and the Pesaran Cross-Sectional Dickey Fuller—CADF- unit-root tests) to establish the integration order of the series. Moreover, we also conduct the Cross-Sectional Dependence tests formulated by Pesaran individually for each variable to inspect the existence of contemporaneous correlation within countries. Finally, to confirm the absence of spurious regressions during the estimations we also conduct Kao’s residual-based cointegration tests.

The unit-root analysis with first and second-generation tests (Supplementary Appendix B, Supplementary Tables B1, B2, B3, B4, B5, B6) confirms that the order of integration is I(1) for the carbon monoxide emissions, economic activity indexes, the total cases of Covid-19, and the controls of unemployment and inflation. These results apply to the full sample and pre covid era. We detected one exception to this general pattern in the post covid era, wherein the Pesaran CADF unit-root tests with cross-sectional dependence were not feasible to perform for some of the variables. As this test requires a larger sample which is not possible with the current data structure that is scattered across two groups, pre-and post-Covid.

Since the variables follow I(1) integration level and to avoid possible model misspecification under imbalanced panel data^47^. We use residual-based and second-generation Westerlund tests and report their outcomes in Supplementary Tables B7, B8, B9, B10. The results confirm the cointegrating relationships between the variables (all sets of covariates included). This applies to the full sample, pre, and post-sample periods. We perform also Pesaran’s Cross-Sectional Dependency tests for each variable (Supplementary Table B11) and the test results confirm the existence of cross-sectionally dependence of all variables.

For model selection, we conduct the Breush-Pagan Lagrange Multiplier tests for heterogeneous effects to detect the existence of non-constant effects in the residuals. After we confirmed the existence of such heterogenous effects (Supplementary Table C1), we then conducted the Hausman test for model selection (Supplementary Table C2), in which we confirmed that a panel fixed effects specification is more convenient and consistent than a random effect. Our fixed effects specification can be subject to issues such as serial correlation, heteroskedasticity, and cross-sectional dependency. We use Huber/White/Sandwich estimators discussed in^48,49^ for autocorrelation and heteroskedasticity along with the Driscoll-Kraay estimators that are robust to prior model biases.

We do not employ the Dynamic Ordinary Least Squares (DOLS) or the Fully Modified Ordinary Least Squares (FMOLS) due to the strong presence of cross-sectional dependence in the variables (see Supplementary Table B11) and missing values. Following^50,51^ using prior methodologies will create biased estimates. As shown in Table 2, our sample involves the top largest economies (following the World’s bank ranking by GDP size^52^).

**Table 2:** World Bank’s ranking of Economies by GDP.

Code	Country
US	United States
JP	Japan
DE	Germany
IN	India
GB	United Kingdom
FR	France
IT	Italy
BR	Brazil
CA	Canada

*Note:* Due to the lack of information from multiple variables of China, it was excluded from this selection. *Source:* World Bank (2019).

### Data description

Table 3 describes the statistical moments of the variables^53–55^ are the main sources of information in our study (See Appendix A, Supplementary A1 for details).

**Table 3:** Global statistics of the sample.

Variable	Type	Mean	Std. Dev	Min	Max	Variable
Carbon Monoxide Emissions	Overall	2.990452	2.294078	0.1	15.5081	N = 465
	Between		2.04175	0.2890212	7.379925	n = 9
	Within		1.213353	0.5970746	15.13745	T = 51.67
EAI	Overall	102.7637	8.878652	79.07644	136.72	N = 508
	Between		8.215709	98.7122	124.1863	n = 9
	Within		3.360363	82.2978	115.2974	T = 56.44
Total cases Covid-19	Overall	1.60e + 07	2.11e + 07	1	1.03e + 08	N = 341
	Between		1.37e + 07	693,119.1	4.72e + 07	n = 9
	Within		1.67e + 07	− 3.12e + 07	7.17e + 07	T bar = 37.8
UE	Overall	6.90813	3.246545	2.2	23.52	N = 508
	Between		2.9773	2.815094	10.70833	n = 9
	Within		1.712054	0.4997966	22.84457	T = 56.44
CPI	Overall	128.0452	51.2197	94.19368	298.99	N = 508
	Between		49.92666	100.8889	257.2794	n = 9
	Within		10.76997	102.0297	169.7558	T = 56.444

*Source:* Own elaboration (2021).

The average carbon monoxide emissions are 2.99 µg per cubic meter of air µg/m^3^, while the Economic Activity Index on average has a value of 102.76, indicating that for the sample between 2014 and 2020, the trend is upward than the unity for the indexes in the countries of analysis, see Table 4. Total cases of Covid-19, on the other hand, have been growing exponentially each month, the unemployment rate on average is 6.9% and the inflation measured by the consumer price index has been growing up reflecting an average value of 128.04 in the inflation index.

**Table 4:** Country level statistics.

ID	Statistic	CO	EAI	Total Cases of Covid-19	UE	CPI
Brazil	Obs	59	60	37	60	60
	Mean	3.830674	99.64846	1.90e + 07	10.70833	120.2091
	Min	1.852273	91.86034	2	4.3	94.19368
	Max	6.79	101.8725	3.70e + 07	14.7	149.7836
	SD	1.285116	1.615178	1.21e + 07	2.614451	16.3577
Canada	Obs	59	62	38	62	62
	Mean	1.462164	99.71908	693,119.1	6.458065	106.0584
	Min	0.8821429	88.26471	3	4.9	98.40528
	Max	2.2	101.1247	1,584,116	13.4	121.2251
	SD	0.2712541	1.854995	577,655.8	1.315301	6.461008
Germany	Obs	16	26	38	26	26
	Mean	3.359299	98.7122	1.21e + 07	3.411538	111.0493
	Min	1.6	90.98957	5	3	105.0175
	Max	5.653571	100.64	3.79e + 07	3.9	122.2204
	SD	1.171069	1.864828	1.42e + 07	0.3755816	5.49475
France	Obs	56	58	38	58	58
	Mean	1.213297	99.54232	1.37e + 07	9.012069	103.4866
	Min	0.1	79.07644	5	7.1	98.86
	Max	2.878947	101.7627	3.85e + 07	10.5	113.38
	SD	0.978935	3.087311	1.11e + 07	1.052329	3.71848
India	Obs	50	59	38	59	59
	Mean	7.379925	99.72229	2.55e + 07	7.583559	122.197
	Min	4.986547	83.55847	1	3.37	102.1358
	Max	11.91111	102.0299	4.47e + 07	23.52	145.9832
	SD	1.657325	3.150527	1.74e + 07	3.228306	13.95595
Italy	Obs	56	67	38	67	67
	Mean	0.2890212	99.54188	8,872,558	10.39403	103.0504
	Min	0.1	86.42143	2	7.9	99.34884
	Max	0.8228682	101.1739	2.55e + 07	12.6	113.9
	SD	0.2405976	2.483372	9,239,691	1.307076	3.466068
Japan	Obs	52	53	38	53	53
	Mean	3.549107	100.0734	6,548,140	2.815094	100.8889
	Min	1.969231	92.51748	15	2.2	99.36371
	Max	5.770513	101.1303	3.30e + 07	3.6	103.3342
	SD	0.7124973	1.291356	1.01e + 07	0.3938939	1.006946
United Kingdom	Obs	57	58	38	58	58
	Mean	3.248914	99.54424	1.06e + 07	4.42931	106.6379
	Min	1.017904	82.51848	2	3.5	99.2
	Max	6.494194	100.9154	2.43e + 07	5.7	122.3
	SD	1.360595	2.914089	9,799,068	0.6431852	6.395937
United States	Obs	60	65	38	65	65
	Mean	3.361105	124.1863	4.72e + 07	4.693846	257.2794
	Min	2.226053	112.17	8	3.5	234.747
	Max	15.5081	136.72	3.72e + 07	13.2	298.99
	SD	2.027132	7.046192	3.24e + 07	1.589701	19.54877
Total	Obs	465	508	341	508	508
	Mean	2.990452	102.7637	1.60e + 07	6.90813	128.0452
	Min	0.1	79.07644	1	2.2	94.19368
	Max	15.5081	136.72	1.03e + 08	23.52	298.99
	SD	2.294078	8.878652	2.11e + 07	3.246545	51.2197

*Note:* Sample from January 2014 to January 2023.

*Source:* Own elaboration (2023).

The country-level statistics report that U.S. and Japan are the countries with the higher average value in the Economic Activity Index, whereas the most pollutants on average in India. The Covid-19 total cases are superior for the U.S. in comparison to the rest of the countries, and the country with the highest unemployment rate in Brazil followed by Italy.

## Empirical results

Multiple models have been estimated using the specification of (1). We scattered our results into three periods, full sample, pre-covid, and post-covid. These results are reported in Appendix D, Supplementary Table D1, D2, D3, D4.

### Full sample

The baseline estimates using the full sample are presented in Supplementary Table D1, both one-way and two-way fixed effects specifications reveal that Covid 19 cases are statistically significant at a 5% level of significance to explain negative changes in the carbon monoxide emissions. However, these baseline results do possess problems of serial correlation and heteroskedasticity. When we included the two-way fixed effects specification (TWFE), the cross-sectional dependency problem disappears. Nevertheless, we proceed to apply the robust standard errors and compare them with the performance of the Driscoll-Kraay estimators to validate the results under the influence of serial correlation and heteroskedasticity (and also potentially cross-sectional dependence).

In our second estimation (Supplementary Table D2) accounting for the serial correlation and heteroskedasticity problems it is noted that the statistical significance of the Covid-19 variable disappears in the full sample. Moreover, the economic activity indexes and the controls are not significant. The Driscoll-Kraay estimates on the contrary report only a change in the statistical significance of inflation.

### Pre and post Covid-19

As the full sample analysis may not be accurate to reflect discrepancies between pre and post-Coid-19. We segment the analysis into pre and post covid 19. The results accounting for serial correlation, heteroskedasticity, and cross-sectional dependency are presented in Supplementary Table D3. The economic activity index is significant in all linear and nonlinear specifications with a 5% level of significance, implying that the N-shape Kuznets curve best describes the dynamics of economic production and carbon monoxide emissions before the outbreak of Covid-19. This result is also consistent with the Driscoll-Kraay estimates.

We move forward to inspecting the covid era (Supplementary Table D4: October 2019 to January 2023). There is some evidence of the Kuznets N-shape curve in the Driscoll-Kraay estimates at a 10% level of significance in this period, but the general pattern is that there is a reduction in the statistical significance and magnitude of all the variables. In fact, in the clustered TWFE estimates, none of the variables is significant. We interpret these results as a rupture in the traditional dynamics of economic production and contamination since the statistical significance is affected severely, remarkably even when there is evidence of the N-shape type of the Kuznets relationships, the statistical significance is not as strong as it was in the pre-covid sample (Supplementary Table D3). Furthermore, the covid variable of total cases is not significant under a 95% level of confidence across the estimates.

**Figure 1:**
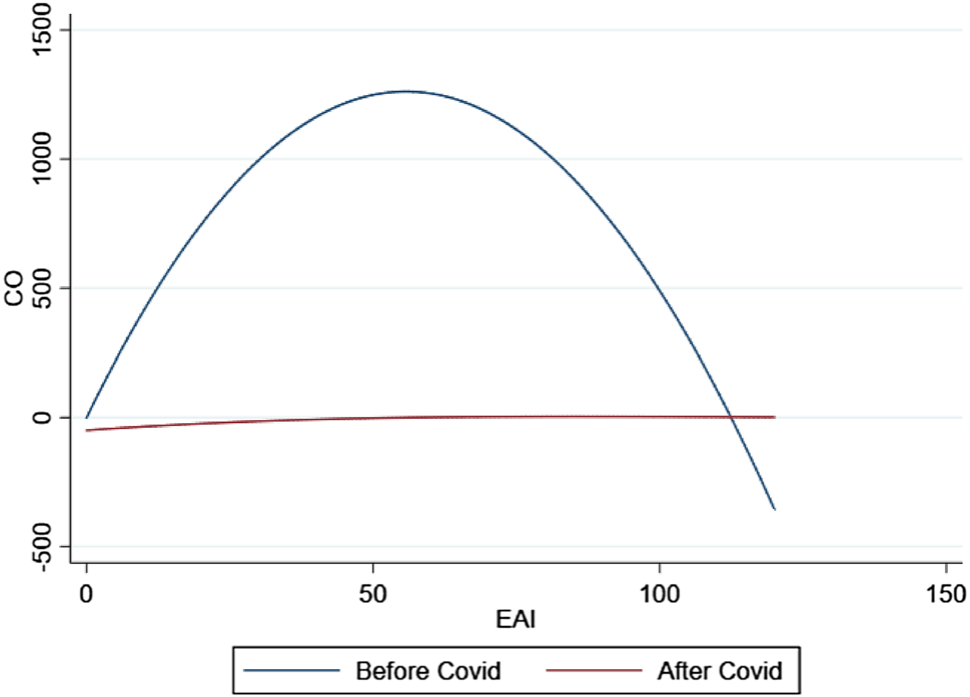
Carbon Monoxide Emissions and Economic Activity: Linear projections of the estimated models.* Source:* Own Elaboration (2023).

To check the robustness of our results, we repeat our estimations after replacing the total cases of Covid 19 variable with another proxy of the covid effects measured as the ratio of monthly total deaths by covid over total infections. We also replace the dependent variable, carbon monoxide emissions, with the first principal components score of multiple pollutants (Carbon monoxide emissions, particulate matter in 2.5 microns –PM_2.5_–, particulate matter in 10 microns –PM_10_–, sulfur dioxide –SO_2_– and nitrogen dioxide –NO_2_–). The new results resemble the primary findings (Supplementary Tables E1 and E2). The N-shape Kuznets curve in the period before the covid era is statistically significant under a 95% level of confidence. This significance disappears, barely significant under a 10% level of confidence in the post-covid (after October 2019). The shape of the Ambiental Kuznets curve changes across the models denoting the eras of pre and post Covid-19 as shown in Fig. 1.

## Conclusion

The study examines the short-run and long-run effects of pre-and post-Covid 19 eras, using the approach of the Ambiental Kuznets curve. The study uses both Driscoll-Kraay and Two-Way Fixed Effects estimators. The result shows that the economic activity index is significant in all linear and nonlinear specifications with a 95% level of significance, implying that the N-shape Kuznets curve best describes the dynamics of economic production and carbon monoxide emissions pre- Covid 19 era. However, in the post-Covid 19 era, our results show that the relationship between economic production and carbon monoxide emissions (air contamination) is no longer significant and does not follow the EKC theory. The study recommends that government and various stakeholders should pursue policies targeting the impact of environmental pollution (carbon monoxide) on economic activity outside the emergence of covid 19.

### Supplementary Information

Supplementary Information 1.

Supplementary Information 2.

Supplementary Information 3.

Supplementary Information 4.

## Data Availability

Requests for data should be addressed to Oduniyi.
