# Supplementary material for: Testing the theory of Kuznet curve on environmental pollution during pre- and post-Covid-19 era
Source: Sci Rep. 2023 Aug 8;13:12851. doi: 10.1038/s41598-023-38962-5 (PMC10409723; doi:10.1038/s41598-023-38962-5)
Supplement: Supplementary file 3 — Supplementary Information 3. [file 41598_2023_38962_MOESM3_ESM.pdf]

```
1   **
2   ** M&S Research hub.
3   **
4
5   clear all
6
7   use "C:\Back up PC My Documents\STATA VER 14\M&S Research Hub\Research Proyects\Third
    Project CO2 Emissions\Data\Stata\data_combined.dta"
8
9
10  ** Drop china and rename total cases variables
11  keep if Country!="CN"
12  encode Country, gen(ID)
13  rename total_cases totalcases
14
15  * Time formatting and month generation
16  gen Time = date(DATE, "YMD")
17  format %td Time
18
19  gen Month = month(Time)
20  gen Year = year(Time)
21
22  gen Time_month = ym(Year,Month)
23  format date %tm
24
25  xtset ID Time_month, m
26  tsfill
27
28  ** Dummy for initial periods
29  gen D_Covid=0
30  replace D_Covid=1 if totalcases!=.
31
32  ** Replacing 0 for missing values of covid to extend the data
33  replace totalcases=0 if totalcases==.
34
35  ** Replacing 0 for missing values of covid deaths to extend the data.
36  replace total_deaths=0 if total_deaths==.
37
38  ** Generation of variables
39
40  xtset ID Time_month, m
41
42  gen EAI_sq=EAI*EAI
43  gen EAI_cu=EAI_sq*EAI
44  gen d_co=d.co
45  gen d_UE=d.UE
46  gen d_CPI=d.CPI
47  gen new_cases=d.totalcases
48  gen new_deaths=d.totalcases
49      ** Seasonal dummy variables
50  tab Year, gen(y)
51  tab Month, gen(m)
52      ** Interacted vars with Covid
53  gen int_EAI_covid = EAI*totalcases
54  gen int_EAI_sq_covid = EAI_sq*totalcases
55  gen int_EAI_cu_covid = EAI_cu*totalcases
56  gen int_UE_covid = UE*totalcases
57  gen int_CPI_covid = UE*totalcases
58      ** Interacted vars with Covid Dummy
59  gen int_EAI_D_covid = EAI*D_Covid
60  gen int_EAI_sq_D_covid = EAI_sq*D_Covid
61  gen int_EAI_cu_D_covid = EAI_cu*D_Covid
62  gen int_UE_D_covid = UE*D_Covid
63  gen int_CPI_D_covid = UE*D_Covid
64      ** logarithms
65  gen ln_co = ln(co)
66  gen ln_EAI = ln(EAI)
67  gen ln_EAI_sq = ln_EAI^2
68  gen ln_EAI_cu = ln_EAI^3
69  gen ln_CPI = ln(CPI)
70  gen ln_UE = ln(UE)
71  gen ln_totalcases= ln(totalcases)
72  gen ln_deaths = ln(total_deaths)
73  gen death_infected_ratio = total_deaths/totalcases
74
```

```
** Unit-root analysis FULL SAMPLE.

xtset ID Time_month, m

xtunitroot fisher co, dfuller lags(1)
    xtunitroot fisher d.co, dfuller lags(1)
xtunitroot fisher EAI, dfuller lags(1)
    xtunitroot fisher d.EAI, dfuller lags(1)
xtunitroot fisher UE, dfuller lags(1)
    xtunitroot fisher d.UE, dfuller lags(1)
xtunitroot fisher CPI, dfuller lags(1)
    xtunitroot fisher d.CPI, dfuller lags(1)
xtunitroot fisher totalcases, dfuller lags(1)
    xtunitroot fisher d.totalcases, dfuller lags(1)
xtunitroot fisher total_deaths, dfuller lags(1)
    xtunitroot fisher d.total_deaths, dfuller lags(1)


    * Co:   Nonstationary - I(1)
    * EAI:  Nonstationary - I(1)
    * UE:   Nonstationary - I(1)
    * CPI:  NonStationary - I(1)
    * Covid Cases: NonStationary - I(1)
    * Covid Deaths: NonStationary - I(1)

** Second generation unit-roots
    *pescadf
    *xtcipsm
    *xtcips
    *multipurt
    // findit pescadf
    // findit multipurt
pescadf co, lags(1)
    pescadf d.co, lags(1)
pescadf EAI, lags(1)
    pescadf d.EAI, lags(1)
pescadf UE, lags(1)
    pescadf d.UE, lags(1)
pescadf CPI, lags(1)
    pescadf d.CPI, lags(1)
pescadf totalcases, lags(1)
    pescadf d.totalcases, lags(1)
pescadf total_deaths, lags(1)
    pescadf d.total_deaths, lags(1)

    ** CIPS test is restrictive to data without gaps (not applicable)
    //xtcips  co, maxlags(1) bglags(1)
    * Second generation of unit-root test reveals the same.

** Unit-root analysis PRE-COVID. Before 2019m10

xtset ID Time_month, m

xtunitroot fisher co if Time_month<tm(2019m10), dfuller lags(1) trend
    xtunitroot fisher d.co if Time_month<tm(2019m10), dfuller lags(1)
xtunitroot fisher EAI if Time month<tm(2019m10), dfuller lags(1)
    xtunitroot fisher d.EAI if Time_month<tm(2019m10), dfuller lags(1)
xtunitroot fisher UE if Time_month<tm(2019m10), dfuller lags(1)
    xtunitroot fisher d.UE if Time_month<tm(2019m10), dfuller lags(1)
xtunitroot fisher CPI if Time_month<tm(2019m10), dfuller lags(1)
    xtunitroot fisher d.CPI if Time_month<tm(2019m10), dfuller lags(1)
  *Note: not deaths or cases in Covid-era.


    * Co:   Nonstationary - I(1)
    * EAI:  Nonstationary - I(1)
    * UE:   Nonstationary - I(1)
    * CPI:  NonStationary - I(1)
    * Covid Cases: NonStationary - I(1)
    * Covid Deaths: NonStationary - I(1)

** Second generation unit-roots
```

```
150       *pescadf
151       *xtcipsm
152       *xtcips
153       *multipurt
154       // findit pescadf
155       // findit multipurt
156   pescadf co if Time_month<tm(2019m10), lags(1)
157       pescadf d.co if Time_month<tm(2019m10), lags(1)
158   pescadf EAI if Time_month<tm(2019m10), lags(1) trend
159       pescadf d.EAI, lags(1)
160   pescadf UE if Time_month<tm(2019m10), lags(1)
161       pescadf d.UE, lags(1)
162   pescadf CPI if Time_month<tm(2019m10), lags(1)
163       pescadf d.CPI if Time_month<tm(2019m10), lags(1)
164    *Note: not deaths or cases in Covid-era.
165
166
167   ** Unit-root analysis POST-COVID. After 2019m10
168
169   xtset ID Time_month, m
170
171   xtunitroot fisher co if Time_month>tm(2019m10), dfuller lags(1)
172       xtunitroot fisher d.co if Time_month>tm(2019m10), dfuller lags(1) trend
173   xtunitroot fisher EAI if Time_month>tm(2019m10), dfuller lags(2) trend
174       xtunitroot fisher d.EAI if Time_month>tm(2019m10), dfuller lags(2)
175   xtunitroot fisher UE if Time_month>tm(2019m10), dfuller lags(1)
176       xtunitroot fisher d.UE if Time_month>tm(2019m10), dfuller lags(1)
177   xtunitroot fisher CPI if Time_month>tm(2019m10), dfuller lags(1)
178       xtunitroot fisher d.CPI if Time_month>tm(2019m10), dfuller lags(1)
179   xtunitroot fisher totalcases if Time_month>tm(2019m10), dfuller lags(1)
180       xtunitroot fisher d.totalcases if Time_month>tm(2019m10), dfuller lags(1)
181   xtunitroot fisher total_deaths if Time_month>tm(2019m10), dfuller lags(1)
182       xtunitroot fisher d.total_deaths if Time_month>tm(2019m10), dfuller lags(1)
183
184       * Co:   Nonstationary - I(1)
185       * EAI:  Nonstationary - I(1)
186       * UE:   Nonstationary - I(1)
187       * CPI:  NonStationary - I(1)
188       * Covid Cases: NonStationary - I(1)
189       * Covid Deaths: NonStationary - I(1)
190
191   ** Second generation unit-roots
192       *pescadf
193       *xtcipsm
194       *xtcips
195       *multipurt
196       // findit pescadf
197       // findit multipurt
198                   //pescadf co if Time_month>tm(2019m10), lags(1)
199                   //pescadf d.co if Time_month>tm(2019m10), lags(1)
200                   //pescadf EAI if Time_month>tm(2019m10), lags(1) trend
201                   //pescadf d.EAI, lags(1)
202                   //pescadf UE if Time_month>tm(2019m10), lags(1)
203                   //pescadf d.UE, lags(1)
204                   //pescadf CPI if Time_month>tm(2019m10), lags(1)
205                   //pescadf d.CPI if Time_month>tm(2019m10), lags(1)
206   pescadf totalcases if Time_month>tm(2019m10), lags(1)
207       pescadf d.totalcases if Time_month>tm(2019m10),  lags(1)
208   pescadf total deaths if Time month>tm(2019m10), lags(1)
209       pescadf d.total_deaths if Time_month>tm(2019m10), lags(1)
210
211    *Note: Covid era, CS augmented tests do not have enough available periods to conduct the
      tests.
212
213
214
215
216   * CSD test
217       //ssc install xtcdf, replace
218   xtcdf co EAI UE CPI totalcases total_deaths
219   *xtcdf co EAI UE CPI totalcases if Time_month<tm(2019m10)
220   *xtcdf co EAI UE CPI totalcases total_deaths if Time_month>tm(2019m10)
221
222       ** FMOLS and DOLS are unsuitable in the CSD case
223       ** See Herzer Nunnenkamp (2012,p.13) The Effect of Foreign Aid on Income Inequality:
```

```
      Evidence from Panel Cointegration
224
225
226   * Cointegration test.
227   xtset ID Time_month, m
228   tsfill
229
230   xtcointtest kao co EAI EAI_sq EAI_cu UE CPI totalcases
231   xtcointtest kao co EAI EAI_sq EAI_cu UE CPI if Time_month<tm(2019m10)
232   xtcointtest kao co EAI EAI_sq EAI_cu UE CPI if Time_month>tm(2019m10)
233
234   xtcointtest westerlund co EAI EAI_sq EAI_cu UE CPI
235   *xtcointtest westerlund co EAI EAI_sq EAI_cu UE CPI if Time_month<tm(2019m10)
236   *xtcointtest westerlund co EAI EAI_sq EAI_cu UE CPI totalcases if Time_month>tm(2019m10)
237
238
239       * Evidence of cointegration at 10% and 5%
240
241   xtcointtest kao co EAI EAI_sq EAI_cu UE CPI totalcases if Time_month>tm(2019m10)
242
243   * Model
244   ***** co = EAI EAI^2  EAI^3 UE CPI + t + f + u
245   ** output folder cd "C:\Back up PC My Documents\STATA VER 14\M&S Research Hub\Research
      Proyects\Third Project CO2 Emissions\Data\Stata\regression tables 2023"
246
247
248   ** Static equations:
249
250   * Battery of tests to select the best model.
251       //1-Pooled OLS
252   xtreg co EAI EAI_sq EAI_cu UE CPI totalcases , re
253   xttest0
254
255               * Both tests reveals the existence of heterogeneis effects
256       //2- Hausmann tests FE vs RE
257   xtreg co EAI EAI sq EAI cu UE CPI totalcases, re
258   estimates store re1
259
260   xtreg co EAI EAI_sq EAI_cu UE CPI totalcases, fe
261   estimates store fe1
262
263       hausman fe1 re1, sigmamore
264               *Both specifications suggest the inclusion of fixed effects
265
266
267
268   ** Outputs.
269       *FE model oneway and twoway
270   xtreg co EAI EAI_sq EAI_cu UE CPI totalcases, fe
271           **outreg2 using fe_models.xls
272   xtreg co EAI EAI_sq EAI_cu UE CPI totalcases m2-m12, fe
273           **outreg2 using fe_models.xls
274
275   * diagnostics.
276   * oneway model
277   xtreg co EAI EAI_sq EAI_cu UE CPI totalcases, fe
278   xttest3
279   predict u0, e
280   xtcdf u0
281   xtserial co EAI EAI_sq EAI_cu UE CPI totalcases, output
282
283   *twoway model
284   xtreg co EAI EAI_sq EAI_cu UE CPI totalcases m2-m12, fe
285                       ** outreg2 using model1.xls
286   xttest3
287   predict u1, e
288   xtcdf u1
289   xtserial co EAI EAI_sq EAI_cu UE CPI totalcases m2-m12, output
290       ** No CSD, Heteroskedasticity Serial Correlation.
291
292
293   xtreg co EAI EAI_sq EAI_cu UE CPI totalcases m2-m12, fe
294           ** outreg2 using model2.xls
295   xtreg co EAI EAI_sq EAI_cu UE CPI totalcases m2-m12, fe cluster(ID)
296           ** outreg2 using model2.xls
```

```
297   xtscc co EAI EAI_sq EAI_cu UE CPI totalcases m2-m12, fe
298           ** outreg2 using model2.xls
299
300   ** Sub-sample analysis
301       ** Dummy of Covid created at the very first to identify missing obs comming from the
      no covid period
302   xtreg co EAI EAI_sq EAI_cu UE CPI m2-m12 if D_Covid ==0, fe cluster(ID)
303           ** outreg2 using model3.xls
304   xtscc co EAI EAI_sq EAI_cu UE CPI m2-m12 if D_Covid ==0, fe lag(1)
305           ** outreg2 using model3.xls
306
307       ** Covid Era
308   xtreg co EAI EAI_sq EAI_cu UE CPI totalcases m2-m12 if D_Covid ==1, fe cluster(ID)
309           ** outreg2 using model4.xls
310   xtscc co EAI EAI_sq EAI_cu UE CPI totalcases m2-m12 if D_Covid ==1, fe lag(1)
311           ** outreg2 using model4.xls
312
313   **
314   **   PRINCIPAL COMPONENTS OF POLLUTANTS - ROBUSTNESS TESTS
315   **
316
317   * An alternative measure of covid effect death/infected ratio.
318
319   replace death_infected_ratio=0 if death_infected_ratio==.
320
321
322   *co  Carbon Monoxide
323   *no2 Nitron Dioxide
324   *pm10 Particulate matter suspended in air or water (less than 10 um diameter)
325   *pm25 Particular matter suspended in than 2.5 um
326   *so2 sulfure dioxide
327
328   pca co pm25 pm10 so2  no2 //
329   estat anti
330   estat kmo
331   estat residuals
332   estat summarize
333   scoreplot
334   loadingplot
335   screeplot
336
337   ** Generation of score of first component
338   pca co pm25 pm10 so2  no2
339   predict PCA_emissions, score
340
341   xtreg co EAI EAI_sq EAI_cu UE CPI death_infected_ratio  m2-m12, fe
342   xtreg co EAI EAI_sq EAI_cu UE CPI death_infected_ratio  m2-m12, fe cluster(ID)
343   xtscc PCA_emissions EAI EAI_sq EAI_cu UE CPI totalcases m2-m12,fe
344
345   *1 Robustness tests
346   xtreg co EAI EAI_sq EAI_cu UE CPI death_infected_ratio  m2-m12 if D_Covid ==0, fe cluster(
      ID)
347           ** outreg2 using robusttest1.xls
348   xtreg co EAI EAI_sq EAI_cu UE CPI death_infected_ratio  m2-m12 if D_Covid ==1, fe cluster(
      ID)
349           ** outreg2 using robusttest1.xls
350   xtscc co EAI EAI_sq EAI_cu UE CPI death_infected_ratio m2-m12 if D_Covid ==0,fe lag(1)
351           ** outreg2 using robusttest1.xls
352   xtscc co EAI EAI sq EAI cu UE CPI death infected ratio m2-m12 if D Covid ==1,fe
353           ** outreg2 using robusttest1.xls
354
355   *2 Robustness tests
356   xtreg PCA_emissions EAI EAI_sq EAI_cu UE CPI totalcases m2-m12 if D_Covid ==0,fe cluster(
      ID)
357           ** outreg2 using robusttest2.xls
358   xtreg PCA_emissions EAI EAI_sq EAI_cu UE CPI totalcases m2-m12 if D_Covid ==1,fe cluster(
      ID)
359           ** outreg2 using robusttest2.xls
360   xtscc PCA_emissions EAI EAI_sq EAI_cu UE CPI m2-m12 if D_Covid ==0,fe lag(0)
361           ** outreg2 using robusttest2.xls
362   xtscc PCA_emissions EAI EAI_sq EAI_cu UE CPI totalcases m2-m12 if D_Covid ==1,fe
363           ** outreg2 using robusttest2.xls
364
365
366
```

```
367   ** Descriptive statistics
368   xtsum co EAI totalcases UE CPI
369   xtsum co EAI totalcases UE CPI if totalcases>0
370   tabstat co EAI totalcases UE CPI, statistics (n mean min max sd ) by(ID)
371   tabstat co EAI totalcases UE CPI if totalcases>0, statistics (n mean min max sd ) by(ID)
372
373   ** Graphs
374   twoway (function y = -1.635 + 45.73*x - 0.421*x^2 + 0.000128*x^3, range(0 120))(function y
       = -49.6 + 1.569*x - 0.0148*x^2 + 4.39e-05 * x^3, range(0 120)), ytitle(CO) xtitle(EAI)
375
376   twoway fpfitci co EAI, ytitle("Average CO Emissions in micrograms")
377   twoway fpfitci co totalcases , ytitle("Average CO Emissions in micrograms")
378
```
