# Supplementary material for: Testing the theory of Kuznet curve on environmental pollution during pre- and post-Covid-19 era
Source: Sci Rep. 2023 Aug 8;13:12851. doi: 10.1038/s41598-023-38962-5 (PMC10409723; doi:10.1038/s41598-023-38962-5)
Supplement: Supplementary file 4 — Supplementary Information 4. [file 41598_2023_38962_MOESM4_ESM.docx]

**APPENDIX A**

Supplementary Table A1. A detailed list of variables by class of indicators and source.

| Country | ID variable | Name of indicator | Class of Indicator | Source |
| --- | --- | --- | --- | --- |
| US | 1 | Coincident Economic Activity Index for the United States | Economic Activity Indicator | https://fred.stlouisfed.org/series/USPHCI |
| US | 2 | Consumer Price Index for All Urban Consumers: All Items in the U.S. City Average | Inflation Index | <https://fred.stlouisfed.org/series/CPIAUCSL> |
| US | 3 | Unemployment Rate for the United States | Unemployment Rate | <https://fred.stlouisfed.org/series/M0892AUSM156SNBR> |
| US | 4 | Population | Population | <https://fred.stlouisfed.org/series/POPTHM> |
| CHINA | 1 | Leading Indicators OECD: Reference series: Gross Domestic Product (GDP): Normalized for China | Economic Activity Indicator | <https://fred.stlouisfed.org/series/CHNLORSGPNOSTSAM> |
| CHINA | 2 | Consumer Price Index CHINA | Inflation Index | <https://fred.stlouisfed.org/searchresults/?st=China%20unemployment> |
| CHINA | 3 | The Urban Surveyed Unemployment Rate(%) | Unemployment Rate | https://data.stats.gov.cn/english/easyquery.htm?cn=A01 |
| JAPAN | 1 | Leading Indicators OECD: Reference series: Gross Domestic Product (GDP): Normalized for Japan | Economic Activity Indicator | <https://fred.stlouisfed.org/series/JPNLORSGPNOSTSAM> |
| JAPAN | 2 | Consumer Price Index of All Items in Japan | Inflation Index | https://fred.stlouisfed.org/series/JPNCPIALLMINMEI |
| JAPAN | 3 | Unemployment Rate of JAPAN | Unemployment Rate | <https://fred.stlouisfed.org/series/LRHUTTTTJPM156S> |
| JAPAN | 4 | Active Population: Aged 15-64: All Persons for Japan | Population | <https://fred.stlouisfed.org/series/LFAC64TTJPM647S> |
| GERMANY | 1 | Leading Indicators OECD: Reference series: Gross Domestic Product (GDP) | Economic Activity Indicator | <https://fred.stlouisfed.org/series/DEULORSGPNOSTSAM> |
| GERMANY | 2 | Consumer Price Index of All Items in Germany | Inflation Index | <https://fred.stlouisfed.org/series/DEUCPIALLMINMEI> |
| GERMANY | 3 | Harmonized Unemployment Rate: Total: All Persons for Germany | Unemployment Rate | <https://fred.stlouisfed.org/series/LRHUTTTTDEM156S> |
| INDIA | 1 | Leading Indicators OECD: Reference series: Gross Domestic Product (GDP): Normalized for India | Economic Activity Indicator | <https://fred.stlouisfed.org/series/INDLORSGPNOSTSAM> |
| INDIA | 2 | Consumer Price Index: All Items for India | Inflation Index | <https://fred.stlouisfed.org/series/INDCPIALLMINMEI> |
| INDIA | 3 | Unemployment Rate Monthly time series (%): India | Unemployment Rate | <https://unemploymentinindia.cmie.com/> |
| UK | 1 | Leading Indicators OECD: Reference series: Gross Domestic Product (GDP): Normalized for the United Kingdom | Economic Activity Indicator | <https://fred.stlouisfed.org/series/GBRLORSGPNOSTSAM> |
| UK | 2 | Consumer Price Index of All Items in the United Kingdom | Inflation Index | <https://fred.stlouisfed.org/series/GBRCPIALLMINMEI> |
| UK | 3 | Harmonized Unemployment Rate: Total: All Persons for the United Kingdom | Unemployment Rate | <https://fred.stlouisfed.org/series/LRHUTTTTGBM156S> |
| FRANCE | 1 | Leading Indicators OECD: Reference series: Gross Domestic Product (GDP): Normalized for France | Economic Activity Indicator | <https://fred.stlouisfed.org/series/FRALORSGPNOSTSAM> |
| FRANCE | 2 | Consumer Price Index of All Items in France | Inflation Index | <https://fred.stlouisfed.org/series/FRACPIALLMINMEI> |
| FRANCE | 3 | Harmonized Unemployment Rate: Total: All Persons for France | Unemployment Rate | <https://fred.stlouisfed.org/series/LRHUTTTTFRM156S> |
| ITALY | 1 | Leading Indicators OECD: Reference series: Gross Domestic Product (GDP): Normalised for Italy | Economic Activity Indicator | <https://fred.stlouisfed.org/series/ITALORSGPNOSTSAM> |
| ITALY | 2 | Consumer Price Index of All Items in Italy | Inflation Index | <https://fred.stlouisfed.org/series/ITACPIALLMINMEI> |
| ITALY | 3 | Harmonized Unemployment Rate: Total: All Persons for Italy | Unemployment Rate | <https://fred.stlouisfed.org/series/LRHUTTTTITM156S> |
| BRAZIL | 1 | Leading Indicators OECD: Reference series: Gross Domestic Product (GDP): Normalised for Brazil | Economic Activity Indicator | <https://fred.stlouisfed.org/series/BRALORSGPNOSTSAM> |
| BRAZIL | 2 | Consumer Price Index: All Items for Brazi | Inflation Index | <https://fred.stlouisfed.org/series/BRACPIALLMINMEI> |
| BRAZIL | 3 | Brazil Unemployment Rate | Unemployment Rate | <https://www.investing.com/economic-calendar/brazilian-unemployment-rate-411> |
| CANADA | 1 | Leading Indicators OECD: Reference series: Gross Domestic Product (GDP): Normalised for Canada | Economic Activity Indicator | <https://fred.stlouisfed.org/series/CANLORSGPNOSTSAM> |
| CANADA | 2 | Consumer Price Index: Total, All Items for Canada | Inflation Index | <https://fred.stlouisfed.org/series/CPALCY01CAM661N> |
| CANADA | 3 | Harmonized Unemployment Rate: Total: All Persons for Canada | Unemployment Rate | <https://fred.stlouisfed.org/series/LRHUTTTTCAM156S> |

Note: The table contains all the name of the variables used for the regression analysis all-in monthly periodicity. It is noted that China was deleted from the analysis due to the lack of information in these variables and missing observations in the same period of the analysis. Source. Own Elaboration (2023).

**APPENDIX B.**

Supplementary Table B1. Fisher-type unit-root Augmented Dickey-Fuller tests (Full Sample).

| Variable | Test |  | Statistic | p-value | Decision |
| --- | --- | --- | --- | --- | --- |
| Carbon Monoxide Emissions | Inverse chi-squared (18) | P | 23.9996 | 0.1550 | Non-stationary |
|  | Inverse normal | Z | -1.2193 | 0.1114 |  |
|  | Inverse logit t(49) | L | -1.2495 | 0.1090 |  |
|  | Modified inv. chi-squared | Pm | 0.9999 | 0.1587 |  |
| Difference Carbon Monoxide Emissions | Inverse chi-squared (18) | P | 165.5224 | 0.0000 | Stationary |
|  | Inverse normal | Z | -8.1934 | 0.0000 |  |
|  | Inverse logit t(49) | L | -14.8246 | 0.0000 |  |
|  | Modified inv. chi-squared | Pm | 24.5871 | 0.0000 |  |
| Economic Activity Index | Inverse chi-squared (18) | P | 38.4453 | 0.0034 | Non-stationary |
|  | Inverse normal | Z | -1.5060 | 0.0660 |  |
|  | Inverse logit t(49) | L | -1.6934 | 0.0487 |  |
|  | Modified inv. chi-squared | Pm | 3.4076 | 0.0003 |  |
| Difference Economic Activity Index | Inverse chi-squared (18) | P | 206.0723 | 0.0000 | Stationary |
|  | Inverse normal | Z | -10.5497 | 0.0000 |  |
|  | Inverse logit t(49) | L | -18.9945 | 0.0000 |  |
|  | Modified inv. chi-squared | Pm | 31.3454 | 0.0000 |  |
| UE Rate | Inverse chi-squared (18) | P | 6.2672 | 0.9950 | Non-stationary |
|  | Inverse normal | Z | 3.2912 | 0.9995 |  |
|  | Inverse logit t(49) | L | 3.3732 | 0.9993 |  |
|  | Modified inv. chi-squared | Pm | -1.9555 | 0.9747 |  |
| Difference UE Rate | Inverse chi-squared (18) | P | 159.6069 | 0.0000 | Stationary |
|  | Inverse normal | Z | -10.2033 | 0.0000 |  |
|  | Inverse logit t(49) | L | -14.7903 | 0.0000 |  |
|  | Modified inv. chi-squared | Pm | 23.6011 | 0.0000 |  |
| Inflation Rate (CPI) | Inverse chi-squared (18) | P | 2.2432 | 1.0000 | Non-Stationary |
|  | Inverse normal | Z | 5.9878 | 1.0000 |  |
|  | Inverse logit t(49) | L | 7.0914 | 1.0000 |  |
|  | Modified inv. chi-squared | Pm | -2.6261 | 0.9957 |  |
| Difference Inflation Rate (CPI) | Inverse chi-squared (18) | P | 122.8848 | 0.0000 | Stationary |
|  | Inverse normal | Z | -7.8255 | 0.0000 |  |
|  | Inverse logit t(49) | L | -11.2330 | 0.0000 |  |
|  | Modified inv. chi-squared | Pm | 17.4808 | 0.0000 |  |
| Total Cases Covid-19 | Inverse chi-squared (18) | P | 0.4796 | 1.0000 | Non-Stationary |
|  | Inverse normal | Z | 7.3310 | 1.0000 |  |
|  | Inverse logit t(49) | L | 8.4887 | 1.0000 |  |
|  | Modified inv. chi-squared | Pm | -2.9201 | 0.9983 |  |
| Difference New Total Cases Covid-19 | Inverse chi-squared (18) | P | 121.0843 | 0.0000 | Stationary |
|  | Inverse normal | Z | -8.7221 | 0.0000 |  |
|  | Inverse logit t(49) | L | -11.2077 | 0.0000 |  |
|  | Modified inv. chi-squared | Pm | 17.1807 | 0.0000 |  |

Note: Probabilities for Fisher tests are computed using an asymptotic Chi-square distribution. Newey–West automatic bandwidth selection and Bartlett kernel. Automatic lag length selection based on SIC. Ho: All panels contain unit roots, Ha: At least one panel is stationary, Autoregressive parameter: Panel-specific. Source: Own elaborations (2023).

Supplementary Table B2. Fisher-type unit-root Augmented Dickey-Fuller tests (Pre-covid sample).

| Variable | Test |  | Statistic | p-value | Decision |
| --- | --- | --- | --- | --- | --- |
| Carbon Monoxide Emissions | Inverse chi-squared(16) | P | 27.1471 | 0.0399 | Non-stationary |
|  | Inverse normal | Z | -0.2430 | 0.4040 |  |
|  | Inverse logit t(39) | L* | -0.9445 | 0.1754 |  |
|  | Modified inv. chi-squared | Pm | 1.9705 | 0.0244 |  |
| Difference Carbon Monoxide Emissions | Inverse chi-squared(16) | P | 171.1411 | 0.0000 | Stationary |
|  | Inverse normal | Z | -9.5114 | 0.0000 |  |
|  | Inverse logit t(44) | L* | -16.5359 | 0.0000 |  |
|  | Modified inv. chi-squared | Pm | 27.4253 | 0.0000 |  |
| Economic Activity Index | Inverse chi-squared(16) | P | 31.6482 | 0.0111 | Non-stationary |
|  | Inverse normal | Z | -1.1754 | 0.1199 |  |
|  | Inverse logit t(44) | L* | -1.3899 | 0.0858 |  |
|  | Modified inv. chi-squared | Pm | 2.7662 | 0.0028 |  |
| Difference Economic Activity Index | Inverse chi-squared(16) | P | 114.2317 | 0.0000 | Stationary |
|  | Inverse normal | Z | -6.4656 | 0.0000 |  |
|  | Inverse logit t(44) | L* | -10.7831 | 0.0000 |  |
|  | Modified inv. chi-squared | Pm | 17.3651 | 0.0000 |  |
| UE Rate | Inverse chi-squared(16) | P | 6.9526 | 0.9742 | Non-stationary |
|  | Inverse normal | Z | 3.3444 | 0.9996 |  |
|  | Inverse logit t(44) | L* | 3.5263 | 0.9995 |  |
|  | Modified inv. chi-squared | Pm | -1.5994 | 0.9451 |  |
| Difference UE Rate | Inverse chi-squared(16) | P | 121.6060 | 0.0000 | Stationary |
|  | Inverse normal | Z | -8.6729 | 0.0000 |  |
|  | Inverse logit t(44) | L* | -11.9442 | 0.0000 |  |
|  | Modified inv. chi-squared | Pm | 18.6687 | 0.0000 |  |
| Inflation Rate (CPI) | Inverse chi-squared(16) | P | 3.8844 | 0.9991 | Non-Stationary |
|  | Inverse normal | Z | 3.2035 | 0.9993 |  |
|  | Inverse logit t(44) | L* | 3.2121 | 0.9988 |  |
|  | Modified inv. chi-squared | Pm | -2.1418 | 0.9839 |  |
| Difference Inflation Rate (CPI) | Inverse chi-squared(16) | P | 88.2871 | 0.0000 | Stationary |
|  | Inverse normal | Z | -6.2404 | 0.0000 |  |
|  | Inverse logit t(44) | L* | -8.4080 | 0.0000 |  |
|  | Modified inv. chi-squared | Pm | 12.7787 | 0.0000 |  |

Note: Probabilities for Fisher tests are computed using an asymptotic Chi-square distribution. Newey–West automatic bandwidth selection and Bartlett kernel. Automatic lag length selection based on SIC. Ho: All panels contain unit roots, Ha: At least one panel is stationary, Autoregressive parameter: Panel-specific. Source: Own elaborations (2023).

Supplementary Table B3. Fisher-type unit-root Augmented Dickey-Fuller tests (Post-covid sample).

| Variable | Test | |  | | Statistic | | | p-value | | Decision |
| --- | --- | --- | --- | --- | --- | --- | --- | --- | --- | --- |
| Carbon Monoxide Emissions | Inverse chi-squared(16) | | P | | 12.3085 | | | 0.7225 | | Non-stationary |
|  | Inverse normal | | Z | | 0.0883 | | | 0.5352 | |  |
|  | Inverse logit t(39) | | L* | | 0.2054 | | | 0.5808 | |  |
|  | Modified inv. chi-squared | | Pm | | -0.6526 | | | 0.7430 | |  |
| Difference Carbon Monoxide Emissions | Inverse chi-squared(16) | | P | | 29.7789 | | | 0.0192 | | Stationary |
|  | Inverse normal | | Z | | -1.1041 | | | 0.1348 | |  |
|  | Inverse logit t(34) | | L* | | -1.9221 | | | 0.0315 | |  |
|  | Modified inv. chi-squared | | Pm | | 2.4358 | | | 0.0074 | |  |
| Economic Activity Index | Inverse chi-squared(16) | | P | | 21.9800 | | | 0.1438 | | Non-stationary |
|  | Inverse normal | | Z | | -0.5072 | | | 0.3060 | |  |
|  | Inverse logit t(44) | | L* | | -0.4913 | | | 0.3128 | |  |
|  | Modified inv. chi-squared | | Pm | | 1.0571 | | | 0.1452 | |  |
| Difference Economic Activity Index | Inverse chi-squared(16) | | P | | 121.6731 | | | 0.0000 | | Stationary |
|  | Inverse normal | | Z | | -5.3830 | | | 0.0000 | |  |
|  | Inverse logit t(44) | | L* | | -12.0995 | | | 0.0000 | |  |
|  | Modified inv. chi-squared | | Pm | | 18.6805 | | | 0.0000 | |  |
| UE Rate | Inverse chi-squared(16) | | P | | 28.9766 | | | 0.0241 | | Non-stationary |
|  | Inverse normal | | Z | | -0.6568 | | | 0.2556 | |  |
|  | Inverse logit t(44) | | L* | | -1.3032 | | | 0.0996 | |  |
|  | Modified inv. chi-squared | | Pm | | 2.2940 | | | 0.0109 | |  |
| Difference UE Rate | Inverse chi-squared (16) | | P | | 38.2256 | | | 0.0014 | | Stationary |
|  | Inverse normal | | Z | | -2.7124 | | | 0.0033 | |  |
|  | Inverse logit t(44) | | L* | | -2.7202 | | | 0.0047 | |  |
|  | Modified inv. chi-squared | | Pm | | 3.9290 | | | 0.0000 | |  |
| Inflation Rate (CPI) | Inverse chi-squared (16) | | P | | 43.1804 | | | 0.0003 | | Non-Stationary |
|  | Inverse normal | | Z | | 2.2939 | | | 0.9891 | |  |
|  | Inverse logit t(44) | | L* | | 1.8689 | | | 0.9658 | |  |
|  | Modified inv. chi-squared | | Pm | | 4.8049 | | | 0.0000 | |  |
| Difference Inflation Rate (CPI) | Inverse chi-squared (16) | | P | | 85.7787 | | | 0.0000 | | Stationary |
|  | Inverse normal | | Z | | -3.3726 | | | 0.0004 | |  |
|  | Inverse logit t(44) | | L* | | -7.5302 | | | 0.0000 | |  |
|  | Modified inv. chi-squared | | Pm | | 12.3352 | | | 0.0000 | |  |
| Covid - 19 Total cases | | Inverse chi-squared (18) | | P | | 1.4291 | 1.0000 | | Not stationary | |
|  |  | Inverse normal | | Z | | 4.9769 | 1.0000 | |  |  |
|  |  | Inverse logit t(49) | | L* | | 5.1543 | 1.0000 | |  |  |
|  |  | Modified inv. chi-squared | | Pm | | -2.7618 | 0.9971 | |  |  |
| Difference Covid - 19 Total cases ¿ | | Inverse chi-squared (18) | | P | | 73.4033 | 0.0000 | | Stationary | |
|  |  | Inverse normal | | Z | | -6.0521 | 0.0000 | |  |  |
|  |  | Inverse logit t(49) | | L* | | -6.7112 | 0.0000 | |  |  |
|  |  | Modified inv. chi-squared | | Pm | | 9.2339 | 0.0000 | |  |  |

Note: Probabilities for Fisher tests are computed using an asymptotic Chi-square distribution. Newey–West automatic bandwidth selection and Bartlett kernel. Automatic lag length selection based on SIC. Ho: All panels contain unit roots, Ha: At least one panel is stationary, Autoregressive parameter: Panel-specific. Source: Own elaborations (2023).

Supplementary Table B4. Pesaran’s Cross-Sectional Dependence unit-root tests (full sample).

|  | Z[t-bar] test | P-value | Decision |  |
| --- | --- | --- | --- | --- |
| Carbon Monoxide Emissions | 3.784 | 1.000 | Non-stationary |  |
| Difference Carbon Monoxide Emissions | -3.890 | 0.000 | Stationary |  |
| Economic Activity Index | 0.723 | 0.765 | Non-stationary |  |
| Difference Economic Activity Index | -2.199 | 0.014 | Stationary |  |
| UE Rate | 3.717 | 1.000 | Non-stationary |  |
| Difference UE Rate | -7.199 | 0.000 | Stationary |  |
| Inflation Rate (CPI) | 5.382 | 1.000 | Non-stationary |  |
| Difference Inflation Rate (CPI) | -5.999 | 0.000 | Stationary |  |
| Covid - 19 Total cases | 0.474 | 0.682 | Non-stationary | |
| Difference Covid - 19 Total cases ¿ | -10.210 | 0.000 | Stationary | |

Note: Pesaran's CADF test. Cross-sectional average in first period extracted and extreme t-values truncated. Panel is unbalanced, only standarized Ztbar statistic can be calculated. Augmented by 1 lags (average). Source Own Elaboration.

Supplementary Table B5. Pesaran’s Cross-Sectional Dependence unit-root tests (pre-covid sample).

|  | Z[t-bar] test | P-value | Decision |
| --- | --- | --- | --- |
| Carbon Monoxide Emissions | 3.016 | 0.999 | Non-stationary |
| Difference Carbon Monoxide Emissions | -6.235 | 0.000 | Stationary |
| Economic Activity Index | -1.560 | 0.059 | Non-stationary |
| Difference Economic Activity Index | -2.199 | 0.014 | Stationary |
| UE Rate | 4.931 | 1.000 | Non-stationary |
| Difference UE Rate | -7.199 | 0.000 | Stationary |
| Inflation Rate (CPI) | -0.763 | 0.223 | Non-stationary |
| Difference Inflation Rate (CPI) | -3.868 | 0.000 | Stationary |

Note: Pesaran's CADF test. Cross-sectional average in first period extracted and extreme t-values truncated. Panel is unbalanced, only standarized Ztbar statistic can be calculated. Augmented by 1 lags (average). Source Own Elaboration.

Supplementary Table B6. Pesaran’s Cross-Sectional Dependence unit-root tests (Post-covid sample).

|  | Z[t-bar] test | P-value | Decision |  |
| --- | --- | --- | --- | --- |
| Carbon Monoxide Emissions | - | - | Insufficient Observations |  |
| Difference Carbon Monoxide Emissions | - | - | Insufficient Observations |  |
| Economic Activity Index | - | - | Insufficient Observations |  |
| Difference Economic Activity Index | - | - | Insufficient Observations |  |
| UE Rate | - | - | Insufficient Observations |  |
| Difference UE Rate | - | - | Insufficient Observations |  |
| Inflation Rate (CPI) | - | - | Insufficient Observations |  |
| Difference Inflation Rate (CPI) | - | - | Insufficient Observations |  |
| Covid - 19 Total cases | 1.839 | -5.760 | Non-stationary | |
| Difference Covid - 19 Total cases ¿ | -3.348 | 0.000 | Stationary | |

Note: Pesaran's CADF test. Cross-sectional average in first period extracted and extreme t-values truncated. The panel is unbalanced, only standarized Ztbar statistic can be calculated. Augmented by 1 lags (average). Source Own Elaboration.

Supplementary Table B7. Kao Residual tests of Cointegration (full sample).

| Test for cointegration | Statistic | p-value |
| --- | --- | --- |
| Modified Dickey-Fuller t | -2.5684 | 0.0051 |
| Dickey-Fuller t | 0.4727 | 0.3182 |
| Augmented Dickey-Fuller t | 3.0767 | 0.0010 |
| Unadjusted modified Dickey Fuller t | -2.4370 | 0.0074 |
| Unadjusted Dickey-Fuller t | 0.5333 | 0.2969 |
|  |  |  |

Note: Covid variable included. Same cointegrating vector for the tests, Panel means included, Autoregressive parameter same for all test. Lags: 1.67 (Newey-West), Bartlett’s kernel selection. Ho: No cointegration, Ha: All panels are cointegrated. Source: Own Elaboration (2023).

Supplementary Table B8. Kao Residual tests of Cointegration (pre-covid).

| Test for cointegration | Statistic | p-value |
| --- | --- | --- |
| Modified Dickey-Fuller t | -4.1639 | 0.0000 |
| Dickey-Fuller t | -1.4808 | 0.0693 |
| Augmented Dickey-Fuller t | 0.5380 | 0.2953 |
| Unadjusted modified Dickey Fuller t | -5.0487 | 0.0000 |
| Unadjusted Dickey-Fuller t | -1.7534 | 0.0398 |
|  |  |  |

Note: Covid variable excluded. Same cointegrating vector for the tests, Panel means included, Autoregressive parameter same for all tests. Lags: 2.63 (Newey-West), Bartlett’s kernel selection. Ho: No cointegration, Ha: All panels are cointegrated. Source: Own Elaboration (2021).

Supplementary Table B9. Kao Residual tests of Cointegration (post covid).

| Test for cointegration | Statistic | p-value |
| --- | --- | --- |
| Modified Dickey-Fuller t | -0.7225 | 0.2350 |
| Dickey-Fuller t | -1.3636 | 0.0863 |
| Augmented Dickey-Fuller t | -1.9548 | 0.0253 |
| Unadjusted modified Dickey Fuller t | -0.7298 | 0.2328 |
| Unadjusted Dickey-Fuller t | -1.3678 | 0.0857 |
|  |  |  |

Note: Covid variable included. Same cointegrating vector for the tests, Panel means included, Autoregressive parameter same for all tests. Lags: 0.67 (Newey-West), Bartlett’s kernel selection. Ho: No cointegration, Ha: All panels are cointegrated. Source: Own Elaboration (2021).

Supplementary Table B10. Westerlund Residual tests of Cointegration (full sample).

| Variance ratio Statistic | P-value |
| --- | --- |
| -2.6173 | 0.0044 |

Note: Covid variable omitted due to large scale of missing values. Number of panels 9, Avg. number of periods 51.667, Cointegrating vector: Panel specific. Panel means Included AR parameter: Panel specific. Ho: No cointegration, Ha: Some panels are cointegrated. Source: Own elaboration.

Table B11. Pesaran’s CD-Test for individual variables.

| Variable | CD-test | p-value | average joint T | mean ρ | mean abs(ρ) |
| --- | --- | --- | --- | --- | --- |
| Carbon Monoxide Emissions | + 5.287 | 0.000 | 44.69 | + 0.16 | 0.39 |
| Economic Activity Index | + 28.973 | 0.000 | 46.44 | + 0.75 | 0.75 |
| Unemployment Rate | + 12.274 | 0.000 | 46.44 | + 0.35 | 0.52 |
| Consumer Price Index | + 38.006 | 0.000 | 46.44 | + 0.95 | 0.95 |
| Covid Total Cases | + 50.675 | 0.000 | 83.14 | + 0.94 | 0.94 |

Note: Under the null hypothesis of cross-section independence, CD ~ N (0,1) . P values close to zero indicate data are correlated across panel groups.

**APPENDIX C.**

Supplementary Table C1. Breusch and Pagan Lagrangian multiplier test for heterogenous effects

| Statistic | Value |
| --- | --- |
| chibar2(01) | 4604.11 |
| Prob > chibar2 | 0.0000 |

Note: Full sample estimates. The null hypothesis is the existence of constant effects from the residuals of the pooled regression. A rejection of this null hypothesis indicates the existence of heterogenous effects in the residuals of the pooled. This test is also known as Breusch and Pagan Lagrangian multiplier test for random effects.

Table C2. Hausmann Tests for model selection of FE and RE

| Statistic | Value |
| --- | --- |
| chi2(4) | 95.44 |
| Prob > chi2 | 0.0000 |

Note: Full sample estimates. A rejection of the null hypothesis indicates that fixed-effects specification is more suitable.

**APPENDIX D.**

Supplementary Table D1. One-way and two-way fixed effects Regressions (full sample)

| VARIABLES | CO | CO |
| --- | --- | --- |
|  |  |  |
| EAI | 2.317 | 1.635 |
|  | (1.948) | (1.968) |
| EAI^2 | -0.0230 | -0.0168 |
|  | (0.0183) | (0.0185) |
| EAI^3 | 7.69e-05 | 5.83e-05 |
|  | (5.63e-05) | (5.69e-05) |
| UE | -0.0187 | -0.0142 |
|  | (0.0363) | (0.0363) |
| CPI | -0.0373*** | -0.0331*** |
|  | (0.0100) | (0.0109) |
| Covid Total Cases | -1.92e-08** | -1.96e-08** |
|  | (8.21e-09) | (8.44e-09) |
| Constant | -71.21 | -46.35 |
|  | (68.24) | (68.96) |
| Individual FE | Yes | Yes |
| Time FE | No | Yes |
| Observations | 465 | 465 |
| R-squared | 0.185 | 0.234 |
| Number of ID | 9 | 9 |

Note: Fixed-effects (within) regression. Models exhibit group-wise heteroskedasticity (via Modified Wald test for groupwise heteroskedasticity) with a 1% significance and serial correlation (via Wooldridge test for autocorrelation in panel data). Testing Cross-Sectional Dependence via Pesaran’s tests indicates the absence of strong cross-sectional dependence at a 1% level of significance in the twoway specification. Source Own elaboration.

Supplementary Table D2. Two-way fixed effects Regressions (full sample)

| VARIABLES | | CO (TWFE) | | CO (DK) | |
| --- | --- | --- | --- | --- | --- |
|  | |  | |  | |
| EAI | | 1.635 | | 1.635 | |
|  | | (1.643) | | (1.622) | |
| EAI_sq | | -0.0168 | | -0.0168 | |
|  | | (0.0157) | | (0.0156) | |
| EAI_cu | | 5.83e-05 | | 5.83e-05 | |
|  | | (4.91e-05) | | (4.90e-05) | |
| UE | | -0.0142 | | -0.0142 | |
|  | | (0.0647) | | (0.0381) | |
| CPI | | -0.0331 | | -0.0331** | |
|  | | (0.0179) | | (0.0160) | |
| Total cases | | -1.96e-08 | | -1.96e-08 | |
|  | | (1.19e-08) | | (1.75e-08) | |
| Constant | | -46.35 | | -46.35 | |
|  | | (57.48) | | (55.82) | |
| Individual FE | Yes | | Yes | |  |
| Time FE | No | | Yes | |  |
| Observations | | 465 | | 465 | |
| R-squared | | 0.234 | | 0.2342 | |
| Number of groups | | 9 | | 9 | |

Note: Full sample (including pre and post covid), TWFE have robust standard errors clustered at the country level controlling for autocorrelation and heteroskedasticity in the SE’s, second column represents the Driscoll Kraay estimates.

Supplementary Table D3. Two-way fixed effects Regressions (Pre-Covid sample)

| VARIABLES | CO (TWFE) | CO (DK) |
| --- | --- | --- |
|  |  |  |
| EAI | 45.73*** | 45.73** |
|  | (2.918) | (20.23) |
| EAI_sq | -0.421*** | -0.421** |
|  | (0.0242) | (0.185) |
| EAI_cu | 0.00128*** | 0.00128** |
|  | (6.81e-05) | (0.000563) |
| UE | 0.0631 | 0.0631 |
|  | (0.0902) | (0.0878) |
| CPI | -0.0745 | -0.0745*** |
|  | (0.0505) | (0.0259) |
| Constant | -1,635*** | -1,635** |
|  | (116.9) | (730.6) |
|  |  |  |
| Observations | 319 | 319 |
| R-squared | 0.246 | 0.2458 |
| Number of groups | 8 | 8 |

Note: Sample corresponding to pre covid era, excluding the data of Covid cases. TWFE have robust standard errors clustered at the country level, second column represents the Driscoll Kraay estimates.

Supplementary Table D4. Two-way fixed effects Regressions (Covid sample)

| VARIABLES | CO (TWFE) | CO (DK) |
| --- | --- | --- |
|  |  |  |
| EAI | 1.569 | 1.569* |
|  | (1.701) | (0.819) |
| EAI_sq | -0.0148 | -0.0148* |
|  | (0.0162) | (0.00791) |
| EAI_cu | 4.39e-05 | 4.39e-05* |
|  | (4.95e-05) | (2.49e-05) |
| UE | -0.0781 | -0.0781* |
|  | (0.0430) | (0.0398) |
| CPI | 0.000647 | 0.000647 |
|  | (0.0172) | (0.0185) |
| Total cases | -3.10e-09 | -3.10e-09 |
|  | (6.32e-09) | (1.00e-08) |
| Constant | -49.60 | -49.60* |
|  | (58.72) | (28.56) |
|  |  |  |
| Observations | 146 | 146 |
| R-squared | 0.320 | 0.320 |
| Number of groups | 9 | 9 |

Note: Sample corresponding to covid era, including the data of Covid cases in the sample. TWFE have robust standard errors clustered at the country level, second column represents the Driscoll Kraay estimates.

**APPENDIX E.**

Supplementary Table E1. Robustness tests pre and post covid era TWFE and DK estimates.

| Timming | Pre Covid | Post Covid | Pre Covid | Post Covid |
| --- | --- | --- | --- | --- |
| Estimators | TWFE | TWFE | DK | DK |
| VARIABLES | CO | CO | CO | CO |
| EAI | 45.73*** | 1.323 | 45.73** | 1.323** |
|  | (2.918) | (1.763) | (20.23) | (0.508) |
| EAI_sq | -0.421*** | -0.0121 | -0.421** | -0.0121** |
|  | (0.0242) | (0.0170) | (0.185) | (0.00496) |
| EAI_cu | 0.00128*** | 3.51e-05 | 0.00128** | 3.51e-05** |
|  | (6.81e-05) | (5.20e-05) | (0.000563) | (1.58e-05) |
| UE | 0.0631 | -0.0698 | 0.0631 | -0.0698 |
|  | (0.0902) | (0.0399) | (0.0878) | (0.0429) |
| CPI | -0.0745 | -0.00430 | -0.0745*** | -0.00430 |
|  | (0.0505) | (0.0135) | (0.0259) | (0.00910) |
| death_infected_ratio |  | 2.410 | 0 | 2.410* |
|  |  | (2.385) | (0) | (1.351) |
| Constant | -1,635*** | -42.24 | -1,635** | -42.24** |
|  | (116.9) | (58.82) | (730.6) | (16.58) |
| Observations | 319 | 146 | 319 | 146 |
| R-squared | 0.246 | 0.323 | 0.246 | 0.323 |
| Number of groups | 8 | 9 | 8 | 9 |

Source: Own elaboration.

Supplementary Table E2. Robustness tests pre and post covid era TWFE and DK estimates.

| Timming | Pre Covid | Post Covid | Pre Covid | Post Covid |
| --- | --- | --- | --- | --- |
| Estimators | TWFE | TWFE | DK | DK |
| VARIABLES | PCA_emissions | PCA_emissions | PCA_emissions | PCA_emissions |
|  |  |  |  |  |
| EAI | 13.41*** | 1.621* | - | 1.621* |
|  | (1.117) | (0.800) | - | (0.866) |
| EAI_sq | -0.122*** | -0.0149* | - | -0.0149* |
|  | (0.00920) | (0.00759) | - | (0.00831) |
| EAI_cu | 0.000367*** | 4.42e-05* | - | 4.42e-05 |
|  | (2.55e-05) | (2.33e-05) | - | (2.61e-05) |
| UE | 0.102*** | -0.0613 | - | -0.0613* |
|  | (0.0258) | (0.0409) | - | (0.0307) |
| CPI | -0.0317 | 0.00489 | - | 0.00489 |
|  | (0.0180) | (0.0145) | - | (0.0212) |
| Total cases |  | -1.73e-09 | - | -1.73e-09 |
|  |  | (4.96e-09) | - | (1.24e-08) |
| Constant | -486.9*** | -57.31* | - | -57.31* |
|  | (45.19) | (28.63) | - | (31.18) |
|  |  |  |  |  |
| Observations | 319 | 146 | - | 146 |
| R-squared | 0.172 | 0.279 | - |  |
| Number of groups | 8 | 9 | 8 | 9 |

Note: Due to missing values the correction of the Driscoll Kraay estimators was not feasible in the pre covid era. Nevertheless, we present the estimates for the post covid era which indicates the same pattern as the rests.
